# Supplementary material for: miR-6850 drives phenotypic changes and signaling in high grade serous ovarian cancer
Source: Noncoding RNA Res. 2025 Nov 5;16:104–16. doi: 10.1016/j.ncrna.2025.10.004 (PMC12636381; doi:10.1016/j.ncrna.2025.10.004)
Supplement: Multimedia component 1 [file mmc1.pptx]

## Slide 1
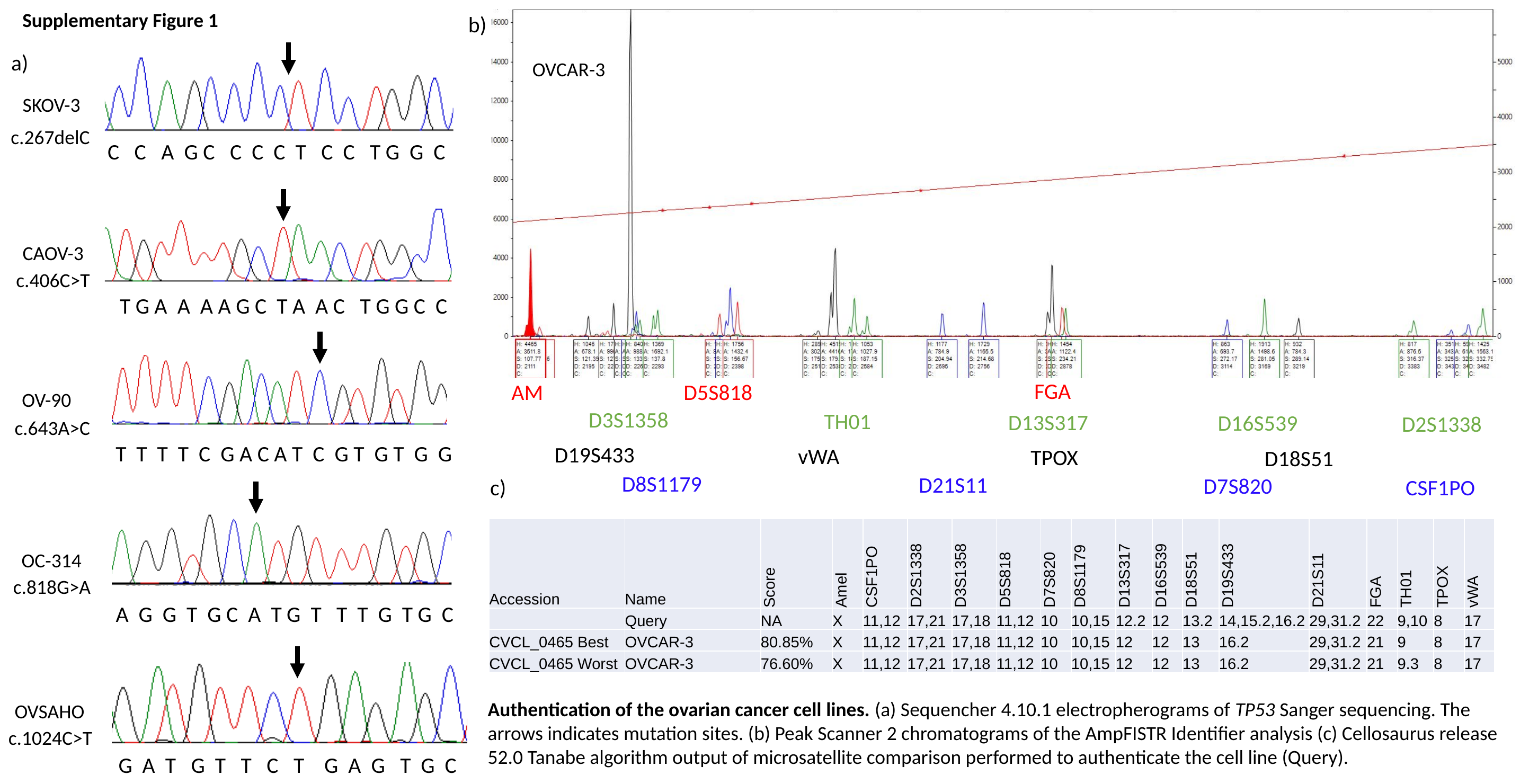

Supplementary Figure 1
b)
FGA
AM
D5S818
D3S1358
TH01
D13S317
D16S539
D2S1338
D19S433
vWA
TPOX
D18S51
D8S1179
D21S11
D7S820
CSF1PO
SKOV-3
c.267delC
C C A G C C C C T C C T G G C
CAOV-3
c.406C>T
T G A A A A G C T A A C T G G C C
OV-90
c.643A>C
T T T T C G A C A T C G T G T G G
OC-314
c.818G>A
A G G T G C A T G T T T G T G C
OVSAHO
c.1024C>T
G A T G T T C T G A G T G C
a)
OVCAR-3
c)
| Accession | Name | Score | Amel | CSF1PO | D2S1338 | D3S1358 | D5S818 | D7S820 | D8S1179 | D13S317 | D16S539 | D18S51 | D19S433 | D21S11 | FGA | TH01 | TPOX | vWA |
| --- | --- | --- | --- | --- | --- | --- | --- | --- | --- | --- | --- | --- | --- | --- | --- | --- | --- | --- |
| | Query | NA | X | 11,12 | 17,21 | 17,18 | 11,12 | 10 | 10,15 | 12.2 | 12 | 13.2 | 14,15.2,16.2 | 29,31.2 | 22 | 9,10 | 8 | 17 |
| CVCL\_0465 Best | OVCAR-3 | 80.85% | X | 11,12 | 17,21 | 17,18 | 11,12 | 10 | 10,15 | 12 | 12 | 13 | 16.2 | 29,31.2 | 21 | 9 | 8 | 17 |
| CVCL\_0465 Worst | OVCAR-3 | 76.60% | X | 11,12 | 17,21 | 17,18 | 11,12 | 10 | 10,15 | 12 | 12 | 13 | 16.2 | 29,31.2 | 21 | 9.3 | 8 | 17 |
Authentication of the ovarian cancer cell lines. (a) Sequencher 4.10.1 electropherograms of TP53 Sanger sequencing. The arrows indicates mutation sites. (b) Peak Scanner 2 chromatograms of the AmpFISTR Identifier analysis (c) Cellosaurus release 52.0 Tanabe algorithm output of microsatellite comparison performed to authenticate the cell line (Query).
